# O último voo: documentos inéditos relativos ao falecimento e ao espólio de Emília Snethlage, 1868-1929

**DOI:** 10.1590/S0104-59702025000100018

**Published:** 2025-05-19

**Authors:** Marco Aurélio Crozariol

**Affiliations:** i Pesquisador, Museu de História Natural do Ceará Prof. Dias da Rocha/Campus Experimental de Educação Ambiental e Ecologia/Universidade Estadual do Ceará. Pacoti – CE – Brasil. marcocrozariol@gmail.com

**Keywords:** Rio Madeira, Mulheres cientistas, Museu Nacional, História da zoologia, Emília Snethlage (1868-1929, Madeira River, Women scientists, National Museum, History of zoology, Emília Snethlage (1868-1929

## Abstract

Emília Snethlage, alemã, veio ao Brasil em 1905 para trabalhar no Museu Paraense Emílio Goeldi, tornando-se uma destacada ornitóloga. Em 1922, foi contratada como naturalista-viajante pelo Museu Nacional. Em setembro de 1929, viajou a Porto Velho para estudar as aves do rio Madeira, até que, na manhã do dia 25 de novembro, foi encontrada morta por uma funcionária do hotel onde estava hospedada. Aqui apresento documentos inéditos, guardados no Museu Nacional, sobre o falecimento da naturalista, entre os quais atestado de óbito, local de sepultamento e espólio encontrados em Porto Velho. Esses documentos escaparam do incêndio de 2018, por estarem em prédio separado.

## Abstract (en)

Emília Snethlage came to Brazil from Germany in 1905 to work at the Emílio Goeldi Museum of Pará [Museu Paraense Emílio Goeldi], going on to become a distinguished ornithologist. In 1922, she started working for the National Museum as a traveling naturalist. In September 1929, she traveled to Porto Velho to study the birds of the Madeira River. On November 25, she was found dead in her hotel room. This study presents unpublished documents from the National Museum concerning Snethlage’s death, some of which were sent there from Porto Velho, including a death certificate, her place of burial, and her estate. These documents escaped the 2018 fire at the museum.

Emília Snethlage (1868-1929), alemã, chegou em Belém do Pará em agosto de 1905, para trabalhar como ornitóloga no Museu Paraense Emílio Goeldi (Hellmayr, 1931; Junghans, 2008). Trabalhou no Museu Goeldi até dezembro de 1921, sendo a primeira mulher a dirigir um museu na América Latina (Estevão, 1938; Lopes, 2008; Junghans, 2011). Em 1922, foi contratada pelo Museu Nacional como naturalista-viajante, cargo que exerceu até novembro de 1929, quando faleceu, no município de Porto Velho, Rondônia, durante expedição para a coleta de aves (Snethlage, 1930; Cunha, 1989). O período em que Emília trabalhou no Museu Goeldi é relativamente bem estudado (Cunha, 1989; Junghans, 2008, 2016; Sanjad et al., 2013; Sanjad, 2019; Santos et al., 2024); porém, os anos em que trabalhou no Museu Nacional são pouco conhecidos no meio acadêmico. Embora publicações recentes tenham trazido informações inéditas sobre Snethlage (Alberto, Sanjad, 2019; Miranda, 2020; Alberto, 2021; Arnegger, Sanjad, 2021; Santos, 2023), aspectos sobre seu falecimento permanecem obscuros. Em contato com a família de Emília, Straube (2017, p.255) ressalta que, “até o momento, nada se conseguiu no sentido de localizar seus restos mortais (Mark Snethlage, in litt., 2013) ... uma situação absolutamente incômoda ... considerando o seu impressionante legado para a ciência”.

Durante uma organização realizada em meados de 2018 no Setor de Ornitologia do Museu Nacional/UFRJ, Departamento de Vertebrados, na Quinta da Boa Vista, Rio de Janeiro, foram descobertos documentos inéditos sobre Emília Snethlage. Entre eles estavam alguns referentes ao falecimento da naturalista, que inclui atestado de óbito, local de sepultamento e espólio encontrados em Porto Velho e no próprio Museu Nacional. Esses documentos são as fontes do presente texto. Todos eles, afortunadamente, sobreviveram ao incêndio que acometeu o palácio na noite de 2 de setembro de 2018, por estarem em prédio separado (Crozariol, 2018), mas estão sem uma numeração de catálogo para que possam ser devidamente referenciados.

## A última expedição: viagem ao rio Madeira

Após ter percorrido boa parte do país ao longo dos quase 25 anos em que vivia no Brasil, Emília Snethlage partiu para estudar as aves do rio Madeira saindo do Rio de Janeiro no dia 25 de setembro de 1929 com destino a Porto Velho, Rondônia, na época estado do Amazonas.

**Figura 1:**
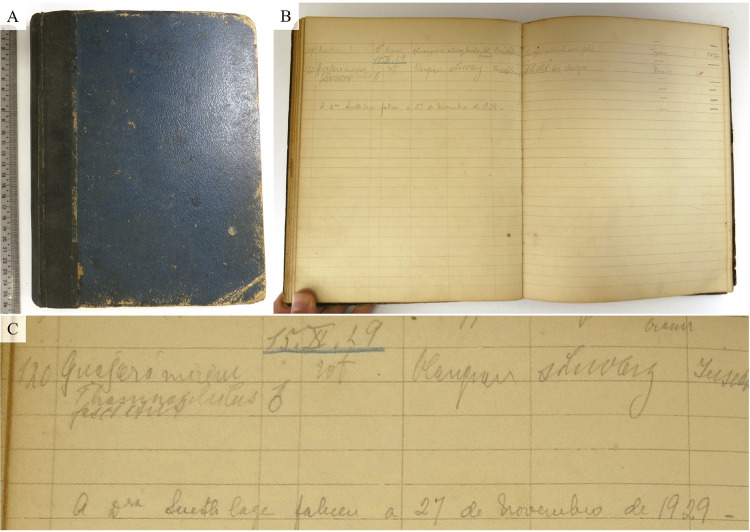
: Caderno de campo de Emília Snethlage, onde se vê (A) a capa; (B) as últimas anotações; (C) detalhe com a 120ª ave da última expedição de sua vida, realizada em Guajará-Mirim, Rondônia, em 15 de novembro de 1929, e a data de falecimento anotada errada no caderno (Fonte: Setor de Ornitologia, Museu Nacional)

Em Porto Velho, hospedou-se no Hotel Brasil, onde atualmente funciona o Teatro Banzeiro, quarteirão delimitado pelas ruas José do Patrocínio, Henrique Dias, Euclides da Cunha e a avenida Rogério Weber e que, na época, pertencia à Madeira-Mamoré Railway Company (Borzacov, 2007, p.35-37). Seu primeiro espécime da expedição, indicado na etiqueta e em seu caderno de coleta^1^ (Figura 1), foi obtido logo no dia seguinte a sua chegada, 22 de outubro. Além de ter realizado algumas viagens curtas pela região, que incluiu a margem esquerda do rio Madeira, Snethlage foi de trem até Guajará-Mirim, tendo saído de Porto Velho entre os dias 12 e 13 de novembro (Snethlage, 1930; Sanjad et al., 2013). Foi em Guajará-Mirim que coletou sua última ave em vida, a 120ª da expedição, conforme indicado em seu caderno de campo para o dia 15 de novembro, apenas dez dias antes de falecer (Figura 1).^2^ Snethlage chegou de volta em Porto Velho no dia 23 de novembro, falecendo dois dias depois, em 25 de novembro de 1929, quando foi encontrada morta na cama por uma funcionária do hotel.

## Falecimento

Raul Andrade, na época delegado de polícia de Porto Velho e encarregado de resolver o incidente, enviou vários documentos e cartas referentes ao falecimento de Snethlage para Edgard Roquette-Pinto (1884-1954), então diretor do Museu Nacional. A primeira delas, em especial, esclarece diversos aspectos ainda desconhecidos sobre o falecimento da naturalista:

ESTADO DO AMAZONAS

DELEGACIA DE POLÍCIA DE PORTO VELHO

Of. n° 62- Em 14 de Dezembro de 1929

Exmo. Snr. Dr. Roquette Pinto

M.D. Diretor do Museu Nacional

Rio de Janeiro

Tendo conhecimento do falecimento da Exma., Sra. Doutora Emilie Snethlage, que se encontrava hospedada no Hotel Brasil, desta cidade, cujo fato ocorreu a 25 do mês passado, tomei todas as medidas que o caso requeria, não só pelos deveres que o cargo me impõe, como por se tratar de pessoa de destaque sem ter a seu lado ninguém conhecido nem de família que se interessasse e, assim, nas averiguações a que procedi me foi comunicado que essa senhora havia permanecido alguns dias na margem esquerda do Rio Madeira, em frente a esta cidade, em pesquisas científicas, voltando a esta cidade, de onde seguiu para Guajará Mirim, estado de Mato-Grosso, e dali voltando no dia 23 à tarde.

De 23 para 24, passou a noite um pouco incomodada, porém durante o dia 24 passou melhor, não tendo, portanto, necessitado de assistência médica, julgando o dono do hotel que se tratasse de qualquer pequeno incômodo.

No dia 25, quando a criada do hotel lhe foi levar o leite, de manhã, encontrou-a morta e na posição descrita no laudo do exame médico, do que me foi dado parte imediata.

Depois de constatar o ocorrido, mandei chamar o Exmo. Sr. Dr. Antonio Magalhães,^3^ diretor do Hospital de Candelária, para proceder o respectivo exame e fazer o levantamento do cadáver. Em seguida, mandei vesti-la convenientemente, mandando fazer o respectivo caixão e conduzir o cadáver para a Igreja desta cidade,^4^ procedendo-se ao seu enterramento às 17 horas.

Por não haver tempo de consultar V. Excia., tomei a liberdade de mandar fazer uma coroa com sua dedicatória, como Diretor do Museu, e de mais empregados, agradecendo em vosso nome, ao Sr. Professor Protazio Silva, as flores naturais que o mesmo depositou sobre a campa.

Para os devidos fins, mandei extrair a certidão de óbito^5^ e o laudo de exame médico, que, junto à presente, bem como um exemplar do jornal *Alto Madeira*,^6^ onde foi publicado a notícia desta lamentável ocorrência.

No dia 26, procedi a arrecadação do espólio, entregando o mesmo ao Sr. primeiro suplente do juiz de direito, em exercício, com uma relação em duas vias, a fim de ser uma remetida a V. Excia., bem como todos os comprovantes das despesas feitas, do que vos envio uma cópia junto, pela qual V. Excia. verá que as despesas foram além da importância requisitada por não me terem fornecido algumas notas a tempo, havendo, portanto, um saldo a meu favor de Rs.57$700, que espero merecerá a aprovação de V. Excia. O referido espólio segue nesta data para Manaus.

O cadáver foi enterrado, provisoriamente, em terreno que se pode perpetuar, pelo que a Prefeitura Municipal cobra a importância de Rs.100$000, no caso que V. Excia. deseje que fique a sepultura perpétua. Sendo assim queira transmitir suas estimadas ordens que serão fielmente cumpridas.

Aproveitando o ensejo para enviar a V. Excia., e aos dignos empregados desse Museu, as minhas condolências pela perda desta digna auxiliar.

Com os meus protestos de alta consideração e estima, desejo a V. Excia.

Saúde e Fraternidade

Raul Andrade [assinado]

Delegado de Polícia.

## Atestado de óbito

**Figura 2:**
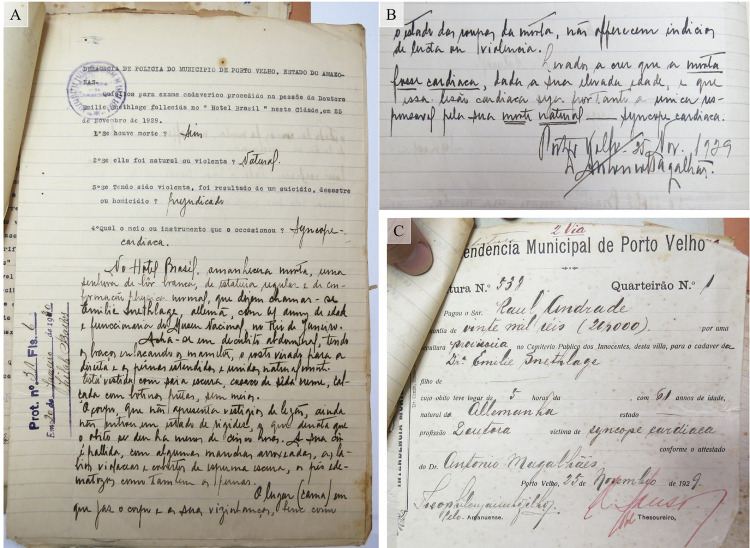
: Atestado de óbito (A e B) e local provisório de sepultamento (C) de Emília Snethlage (Fonte: Setor de Ornitologia, Museu Nacional)

Como mencionado, acompanhando a carta acima transcrita, Raul Andrade enviou ao Museu Nacional o atestado de óbito de Emília Snethlage (Figura 2, A e B), que consiste em apenas uma folha pautada, com uma primeira parte datilografada apresentando informações sobre do que se trata o documento, além de algumas perguntas básicas, respondidas e descritas manuscritamente à tinta:

DELEGACIA DE POLÍCIA DO MUNICÍPIO DE PORTO VELHO, ESTADO DO AMAZONAS.

Quesitos para exame cadavérico procedido na pessoa da Doutora Emilie Snethlage, falecida no Hotel Brasil nesta cidade, em 25 de Novembro de 1929.

1° Se houve morte? [à tinta:] Sim. 2° Se ela foi natural ou violenta? [à tinta:] Natural.

3° Se Tendo sido violenta, foi resultado de um suicídio, desastre ou homicídio? [à tinta:] Prejudicada.

4° Qual o meio ou instrumento que a ocasionou? [à tinta:] Síncope cardíaca.

[Inicia a parte manuscrita]

No Hotel Brasil, amanhecera morta uma senhora de cor branca, de estatura regular e de conformação física normal, que dizem chamar-se Emilie Snethlage, alemã, com 61 anos de idade e funcionária do Museu Nacional, no Rio de Janeiro.

Acha-se em decúbito abdominal, tendo os braços enlaçando os mamelões [mamas], o rosto virado para a direita e as pernas estendidas e unidas naturalmente.

Está vestida com saia escura, casaco de seda creme, calçada com botinas pretas, sem meias.

O corpo, que não apresenta vestígios de lesões, ainda nem entrou em estado de rigidez, o que denota que o óbito se deu há menos de cinco horas. A sua cor é pálida, com algumas manchas arroxeadas, os lábios violáceos e cobertos de espuma escura, os pés edematosos, como também as pernas.

O lugar (cama) em que jaz o corpo e as suas vizinhanças, bem como [passa para a parte traseira da página] o estado das roupas da morta, não oferecem indício de luta ou violência.

Levados a crer que a morta fosse cardíaca, dada a sua elevada idade, e que essa lesão cardíaca seja, portanto, a única responsável pela sua morte natural – síncope cardíaca. [sublinhados no original]

Porto Velho – 25 nov. 1929

Antonio Magalhães.

Segundo a carta de 14 de dezembro enviada por Raul Andrade, Snethlage teria passado mal um dia antes de falecer. Estudando seu espólio encontrado no Hotel Brasil (*vide* adiante), é intrigante o fato de que ela foi encontrada calçando sapatos, porém sem meias, mesmo havendo mais de dez pares delas em sua bagagem. Algumas de suas fotografias conhecidas também mostram-na calçando meias. É possível que Snethlage tenha se sentido mal e, na pressa para buscar ajuda, apenas calçou o sapato sem as meias, acabando por falecer antes de ter tido tempo de sair do quarto.

## Preparação do corpo

O delegado Raul Andrade também se responsabilizou por mobilizar pessoas para a preparação do corpo e do enterro de Snethlage. Alguns documentos indicam os nomes dessas pessoas, os trabalhos por elas executados e os valores envolvidos:

DELEGACIA DE POLÍCIA DA CIDADE DE PORTO VELHO

ESTADO DO AMAZONAS

Demonstração das despesas feitas com o enterramento do cadáver da Doutora Emilie Snethlage, falecida nesta cidade, em 25 de novembro do corrente ano: a saber:

Pago ao Sr. Luiz Paes, pelo feitio de um caixão mortuário 185$000

Idem ao Dr. Antonio Magalhães, pelo exame cadavérico e

serviços profissionais 150$000

Idem ao Sr. Manoel Moraes, de carretos e arrumação do espólio 35$000

Idem a Sra. Da. Maria José, por aceitar e vestir o cadáver 20$000

Idem a Sra. Da. Maria Emilia, por uma coroa mortuária 40$000

Idem ao tabelião [Dimas Weyney], custas do certificado e certidão de óbito 25$000

Idem a Prefeitura Municipal, emolumentos da sepultura 20$000

Idem de dois radiogramas Números 171 e 229 17$700

Rs: 492$700

Encontrado no espólio e gasto com o enterro 35$000

Recebido do Banco do Brasil, por intermédio da The Amazon

River Co, Ltd., remetido pelo Sr. Dr. Roquette Pinto 400$000

435$000

Saldo a meu favor 57$700

Porto Velho, 9 de Dezembro de 1929.

Raul Andrade [assinado]

Delegado de Polícia.

Em outro documento, mais uma carta de Raul Andrade para Roquette-Pinto, consta uma segunda cobrança, uma vez que o Hotel Brasil não havia recebido o pagamento das diárias da hospedagem. Além disso, o estabelecimento cobrava do Museu Nacional uma indenização pelos objetos utilizados por ela quando falecera, pois foram incinerados:

ESTADO DO AMAZONAS

Delegacia de Polícia, em Porto Velho

18 de janeiro de 1930.

Ofício n° 71.

Exmo. Sr. Dr. Roquette Pinto

M.D. Diretor do Museu Nacional

Rio de Janeiro

Dou em meu poder o telegrama de V. Excia., datado a [perdido parte do texto] do corrente, cumprindo-me informar V. Excia. que, efetivamente, a Exma. Sra. Dr. Emílie Snethlage esteve hospedada no Hotel Brasil desta cidade, cujo proprietário me apresentou a conta junto, no valor de (Rs.290$000), Duzentos e noventa mil réis, do que não tomei conhecimento, oficialmente, por ser assunto que me não competia.

Eu e mais algumas pessoas assistimos à incineração das roupas constantes da referida conta, e, como a morte foi repentina, é de prever que não tenha tido tempo para liquidar a sua conta de hospedagem.

Aproveito o ensejo para apresentar a V. Excia., os meus protestos de elevada estima e consideração.

Saúde e fraternidade

Raul Andrade [assinado]

Delegado de Polícia.

Seguem adiante a conta e os seus respectivos valores:

Porto Velho, 25 de novembro de 1929

Sra. Dra. Emilie Snethlage Comp. a M. Souto

3 Dias de Hospedagem^7^ 25$ – 75$000

Roupas de Cama que foram queimadas, sendo;

2 Lençóis 50$000

2 Travesseiros 50$000

1 Cobertor de Lã 75$000

1 Colchão 40$000

Rs......290$000

Importa a presente nota na importância total de Rs. (DUZENTOS E NOVENTA MIL REIS).

[Assinado à tinta] Porto Velho, 16 de Janeiro de 1930

M. Souto

[e outra assinatura não identificada]

## Enterro e local do sepultamento

**Figura 3:**
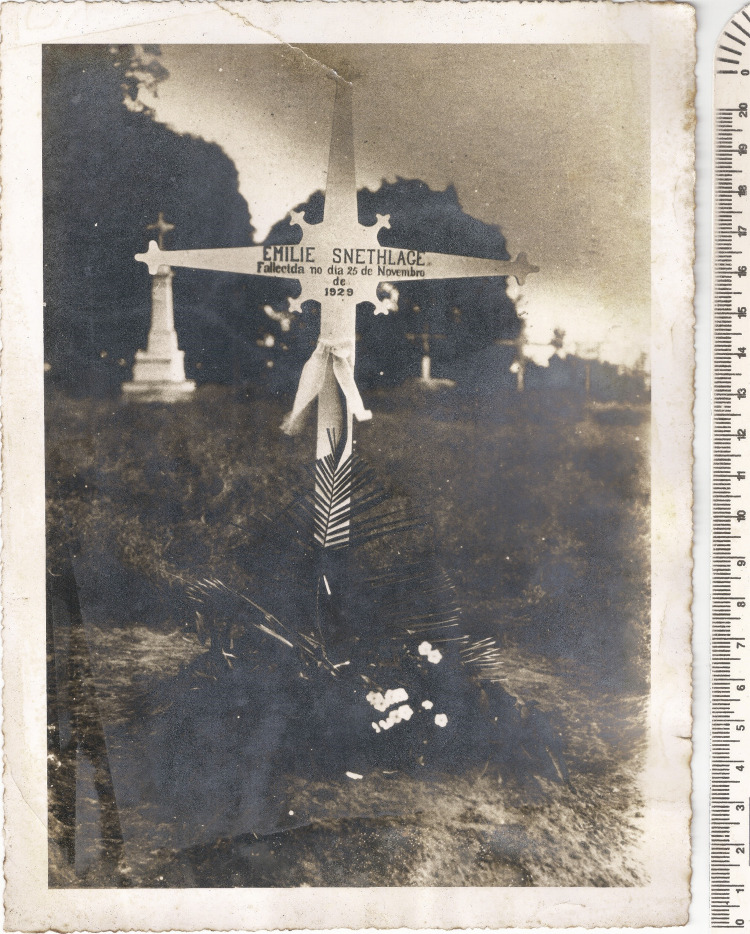
: Local de sepultamento de Emília Snethlage, data desconhecida (Fonte: Setor de Ornitologia, Museu Nacional)

Entre os documentos enviados por Raul Andrade, sem dúvidas um dos mais interessantes é aquele expedido pela Prefeitura de Porto Velho, onde consta o nome do cemitério e o local exato onde Snethlage foi enterrada. Embora se conheçam ao menos duas fotos do seu local de sepultamento, indicado por uma cruz com seu nome (Figura 3), atualmente, a cruz está desaparecida e esse local é desconhecido.

No documento expedido pela prefeitura (Figura 2C) está indicado que Snethlage foi enterrada em sepultura “provisória” no cemitério público dos “Inocentes”,^8^ no quarteirão de “n.1” e sepultura “n.538”. Essa é, até hoje, a informação mais exata sobre o local de sepultamento de Emília Snethlage. Mas, infelizmente, mesmo com essas informações, não foi possível localizar seu túmulo, visto que foi feita uma nova distribuição de quarteirões no cemitério. Uma planta do cemitério daquela época poderia resolver esse caso, porém, em fevereiro de 2019, fiz uma busca nos arquivos da cidade e no próprio cemitério acompanhado de Gilbson Morais, gerente de divisão do departamento dos cemitérios de Porto Velho, mas não foi possível localizar nada.

Vale chamar a atenção para a palavra “provisória”, também mencionada na carta de Raul Andrade a Roquette-Pinto de 14 de dezembro de 1929. Raul Andrade menciona que “a Prefeitura Municipal cobra a importância de Rs.100$000, no caso que V. Excia. deseje que fique a sepultura perpétua”, e no documento de valores envolvidos mostra que a Prefeitura Municipal cobrou apenas 20$000. Acompanham o referido documento notas com a assinatura das pessoas que executaram os serviços e os valores envolvidos; porém, nada consta sobre o valor pago para a Prefeitura. Não foi possível descobrir, portanto, qual quantia foi paga, seja pelo Museu Nacional ou pela família, de forma que há grandes chances de que uma sepultura perpétua não tenha sido escolhida e o corpo da naturalista tenha sido exumado após alguns anos, vindo a se perder.

Apoia a hipótese de túmulo provisório a descrição feita por seu sobrinho, Emil Heinrich Snethlage (1897-1939), que o visitou em 11 de agosto de 1933 e anotou em seu diário que, com “relação às sepulturas com cruz de madeira, é a que estava mais bem conservada, mas era apenas um monte de areia – muito desigual, com a cruz um pouco torta” (Mere et al., 2021, p.116). Em agosto de 1938, Walter Höntsch enviou uma carta a Emil relatando que “queria visitar a sepultura de sua tia, mas não consegui achar em nenhuma parte do cemitério, que já estava totalmente tomado pelo mato. Até andei entre as fileiras das antigas sepulturas, atrás da capela em ruínas – sem sucesso” (Mere et al., 2021, p.116). Com base nesses relatos, as observações realizadas em agosto de 1933 são as últimas conhecidas sobre a cruz e o local identificados como a sepultura de Emília Snethlage.

## Espólio de Emília Snethlage, encontrado no Hotel Brasil

Logo no dia seguinte ao falecimento, a Delegacia de Polícia de Porto Velho apresentou uma relação dos pertences de Snethlage encontrados no Hotel Brasil:

DELEGACIA DE POLÍCIA DE CIDADE DE PORTO VELHO

ESTADO DO AMAZONAS

Relação dos objetos pertencentes à Senhora Doutora Emilie Snethlage, falecida hontem no Hotel Brasil.

Aos vinte e seis dias do mês de Novembro do ano de mil novecentos e vinte e nove, nesta Delegacia de Polícia, presente o Senhor Raul Andrade, Delegado de Polícia, e as testemunhas José Calazans Machado e João Manoel do Nascimento, comigo escrivão *ad hoc* nomeado e abaixo assinado, foi arrolado o espólio da senhora Doutora Emilie Snethlage, falecida repentinamente no Hotel Brasil, nesta cidade, composto de uma maleta, duas malas, um saco de transporte para roupas e dois caixões; sendo pela referida autoridade em presença das testemunhas acima mencionadas, aberto espólio, cujo conteúdo é o seguinte: – MALETA: – Trinta e cinco mil e cem réis, (35$100) cuja importância foi empregada com as despesas do funeral, Cinco Bolivianos, Duas (2) bolsas de couro para senhora; com documentos e miudezas diversas, Um (1) pacote com velinhas de cera; Uma (1) árvore natal (brinquedo), Uma (1) caixa de flandre com acessórios para costuras; Três (3) combinações; Duas (2) escovas para cabelo; Um (1) chapéu; Um (1) cuité;^9^ Dois (2) pentes; Quatro (4) lenços; Um (1) par de meias; Um (1) pince-nez; Quatro (4) livros; Um (1) relógio de algibeira; Um (1) vidro com uma cobra: – MALA N° UM: – Duas (2) caixas de papelão com papéis diversos; Uma (1) caixinha com costuras; Um (1) pacote com Mapas diversos; Três (3) latas com cartuchos para armas; diversas revistas; Um (1) rolo de papel; Um (1) livro de apontamentos. Onze (11) pares de meias grosseiras; Uma (1) tarrafa para apreensão de insetos; Um (1) culote; Um (1) chapéu de pano; Cinco (5) vestidos; Onze (11) combinações; Um (1) tripé de máquina fotográfica: – MALA N° DOIS: Três (3) pacotes com algodão; Um (1) embrulho com estopilha; Três (3) caixas com cartuchos; Um (1) embrulho de chumbo; Um (1) embrulho com drogas; Cinco (5) ratoeiras; Dois (2) pares [segue para página seguinte] de botas; Uma (1) camisa de lona para armas; Uma (1) rede; Um (1) arreio para mala: “SACO”: – Uma (1) máquina fotográfica com diversas chapas; Um (1) pacote com algodão; Duas (2) caixas com pó; Um (1) livro; Cinco (5) latas com cartuchos; Um (1) par de botas; Um (1) par de sapatos; Duas (2) caixas com etiquetas; Um (1) chapéu de pano; Doze (12) ferros diversos; Uma (1) rede; diversas peças de roupas sujas. CAIXÃO N° UM: – Contendo diversos passarinhos preparados. CAIXÃO N° DOIS: – TRÊS (3) espingardas uma vareta para espingarda e um guarda-sol. E nada mais continha no referido espólio, mandou o senhor Delegado de Polícia, lavrar o presente termo, que lido e achado conforme assina com as testemunhas. [Daqui pra frente, manuscrito]. Eu, Pedro Ramos Netto, escrivão *ad hoc*, datilografei e assino.

Raul Andrade

José Calasans Machado

João Manoel do Nascimento

Todo o espólio encontrado no Hotel Brasil foi remetido para Manaus e, de lá, para o Rio de Janeiro, e a data deve ter sido aquela indicada por Raul Andrade para Roquette-Pinto, de 14 de dezembro de 1929, como o próprio Raul Andrade menciona na carta. Não foi possível descobrir a data em que o material chegou em Manaus, mas provavelmente partiu dessa cidade no dia 30 de dezembro, chegando no Rio de Janeiro por volta do dia 21 de janeiro de 1930, quando um memorando foi redigido para Roquette-Pinto. Com esse documento, havia outros dois a ele anexados, com informações sobre o material transportado, os valores cobrados pela empresa Berringer & Cia. e o nome do navio que fez o transporte do espólio: Almirante-Jaceguay.

**Figura 4:**
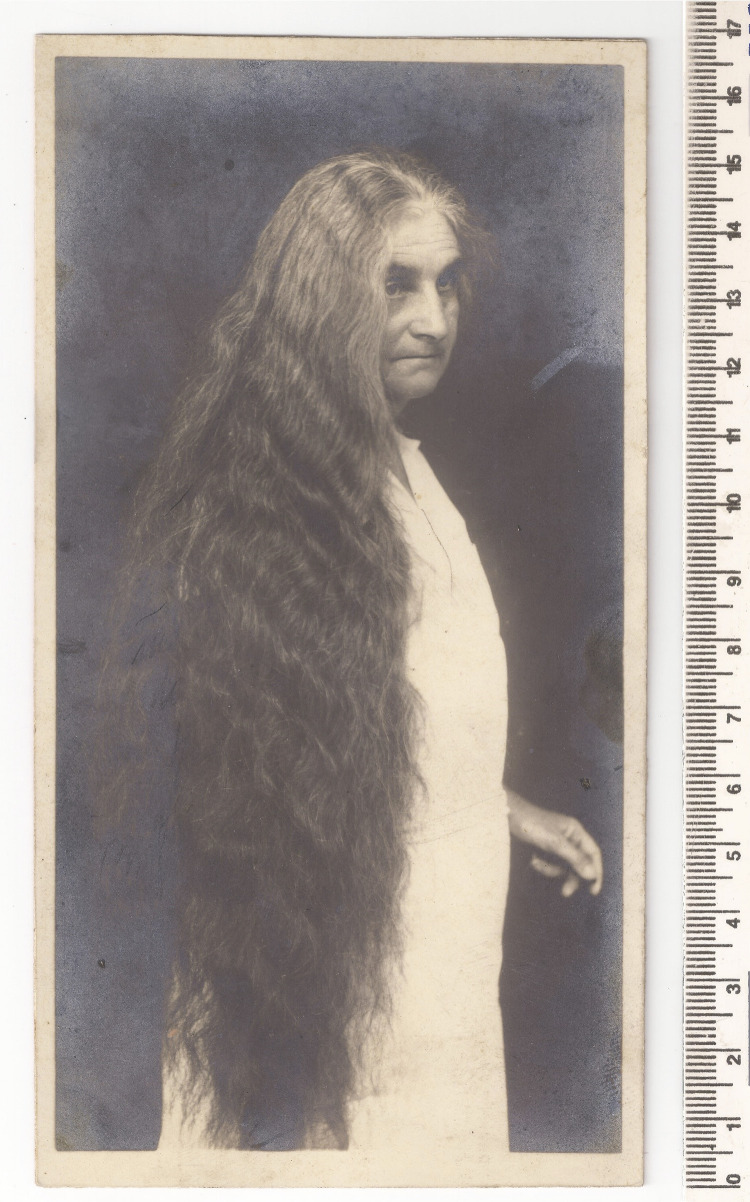
: Única foto conhecida de Emília Snethlage com cabelo solto, provavelmente década de 1920 (Fonte: Setor de Ornitologia, Museu Nacional)

Aqui apenas como uma curiosidade sobre a vida pessoal de Snethlage: foi listado em seu espólio, além de dois pentes, duas escovas de cabelo. Snethlage tinha o cabelo bastante longo, algo ligado possivelmente a sua religião, luterana, e também, como ela mencionava, isso a ajudava na forma de ser tratada, como mulher, em campo (Roquette-Pinto, 1940). Em rara fotografia encontrada juntamente com os documentos aqui apresentados (Figura 4), Snethlage aparece com os cabelos soltos. Ela possuía máquina fotográfica e tripé, também listados no espólio; teria ela mesma feito essa foto ou outra pessoa?

## Espólio de Emília Snethlage, na época, encontrado no Museu Nacional

Tendo Snethlage falecido em expedição, e, portanto, tratando-se de uma situação inesperada, muitos dos seus materiais de trabalho estavam no Museu Nacional, especificamente na Seção de Zoologia, da qual o zoólogo Alípio de Miranda-Ribeiro (1874-1939) era chefe.

Em documento datado de 30 de novembro de 1929, Miranda-Ribeiro encaminhou a Roquette-Pinto uma lista, bastante detalhada, dos “papéis e documentos existentes” na Seção de Zoologia pertencentes a Emília Snethlage. Essa lista também foi assinada por Eduardo May, então naturalista do Museu Nacional. Todas as folhas têm a assinatura de Miranda-Ribeiro rubricada no canto superior esquerdo.

Rio de Janeiro, 30 de novembro de 1929.

Sr. Diretor

De acordo com o ofício de n.19 de 27 último dessa Diretoria, envio juntamente, de acordo com a relação respectiva, em duas vias, um volume contendo todos os papéis e documentos existentes nesta Seção e de propriedade da Sra. Dra. Emilia Snethlage, de saudosa memória e que exerceu aqui as funções de naturalista contratada.

Saúde e Fraternidade.

Miranda Ribeiro

Prof. Chefe da Seção.

LISTA DE PAPÉIS, DOCUMENTOS E LIVROS PARTICULARES DA DOUTORA EMILIE SNETHLAGE, FALECIDA EM PORTO VELHO, RIO MADEIRA EM [não preenchido] DE NOVEMBRO DE 1929.

- - - - - - - - - -

PACOTE N° 1 – 7 Cartas fechadas endereçadas à Doutora N° 1-7

PACOTE N° 2 – 42 Cartas abertas com e sem envelope N° 8-49

PACOTE N° 3 – 16 Cartões e folhetos de anúncio N° 50-65

PACOTE N° 4 – 1 Carl Hegenbeck’s Illustrierte Tier-und

Menschenwelt N° 66

1 Coloured Plates Birds of Ceylon N° 67

1 Monograph of the Birds of Prey N° 68 1 Mittel und Sudamerikanische Amphibien N° 69

1 Tertiare Wirbeltiere – Lorenz Müller N° 70

PACOTE N° 5 – 1 coleção da “A informação Goiana” N° 71a-g

1 coleção da “A informação Goiana” N° 72a-k

1 colecção da “A informação Goiana” N° 73a-h

PACOTE N° 6 – 1 Tabelle zur Aufnahme sudamerikanischer Sprachen N° 74

1 Die Palikur – Indianer und Ihre Nachbarn N° 75

1 Deutsch – Brasilianischer Handels-Kalender 1926 N° 76

1 Beiträge zur Osteologie der Rezenten Krokodilier N° 77

1 Separata ais “Naturwissenschaftlicher Beobachter”. Heft n° 9-12 N° 78

1 Separata “Senckenbergiana” Bd. IV, Heft. 6 N° 79

1 Separata “Senckenbergiana” Bd. VI, Heft. 5/6 N° 80

7 Separatas Zoologischen Anzeiger Bd.

XXXI-2, LVII-2, LVIII-2, LIX-1 N° 81a-g

1 Sonderabdruck aus Journal für Ornithologie LXXV, Heft 2 N° 82

1 Sonderabdruck Naturwissenschaftlicher Beobachter, Heft N° 15/16 N° 83

1 Beiträge zur Brasilianischen Oologie E. Snethlage und Karl Schreiner N° 84

1 Einige neue Ameisen aus Brasilien. Von Th. Borgmeier N° 85

1 Die Flüsse Iriri und Curupa im Gebiete des Xingú N° 86 a-c

1 Die Vogelstimmenforschung als Wissenschaft N° 87

1 Journal für Ornithologie. Heft 3. LXXIII 1925. N° 88

1 Journal für Ornithologie. Heft 2. LXXIII 1925 N° 89

1 Von unseren Reptilien und Lurchen N° 90

1 Der Halsbandfliegenfänger in unterientunde N° 91

1 Blätter für Aquarien und Terrarientunde N° 92

1 Aus des Zeitschrift für Ethnologie N° 93

9 Der eigene Weg N°94-102

PACOTE N° 7 – 1 A Study of the Neotropical Finches of Genus Spinus N° 103

1 Nota de publicador – popular Handbook of Indian Birds N° 104

1 Nota biográfica Frederico Augustus Lucas. N° 105

PACOTE N° 7 – 1 A Year’s Program for Birds Protection N° 106

1 Beiträge zur Brasilianischen Oologie N° 107

1 Prospecto de Naturschutz N° 108

1 Prospecto Birds of New Mexico N° 109

1 Catálogo de livros Científicos Bowes & Bowes. N° 110

1 Catálogo de livros de História Natural Lowe Brothers. N° 111

1 Catálogo de livros Fisiologia – zoologia N° 112

1 Ornithologische Monatsberichte N° 113

1 List of Types of Birds – Canergie Museum. N° 114

1 Prospecto de Nature Stories – American Museum N° 115

1 Prospecto de A Serie of Popular Lectures N° 116

1 Prospecto de Guide to the Birds of Europe and North Africa N° 117

1 Prospecto de British Birds N° 118

1 The Borders and Beyond N° 119

1 The Status of the Great White Heron & Ernest G. Holt. N° 120

1 The Most Valuable Bird in the World R.C. Murphy N° 121

1 The House (Wrens [à caneta]) of the Genus Troglodytes (Sep. A.M.N.H.) N° 122

1 The Birds of the Tambelan Islands – H. C. Oberholser N° 123

1 Notes on Birds collected on Pulo Taya N° 124

1 A Revision of the Sub-species of the White Collard Kingfisher N° 125

1 Notes on Dr. W.L. Abbott’s second collection from Simalur Island, Western Sumatra. N° 126

PACOTE N° 8 – 1 The common Ravens of North America Harry C. Oberholser N° 127

1 New Light on the Status of Empidonax traillii (Audubon) Oberholser. N° 128

1 A Revision of the Sub-species of Passerculus rostratus (Cassin) Oberholser N° 129

1 Description of a new sub-species of Cyanolaemus clemenciae Oberholser N° 130

1 Description of a new Lanius from Lower California Oberholser N° 131

1 Description of an interesting new Junco from L. Calif. Oberholser N° 132

1 Description of a new sub-species of Pipilo fuscus N° 133

13 Notas (sobre [à caneta]) Ornithoilogia- rep. do Journal of the Washington Academy of Sciences N°134a-m

1 Retrospect (prospectus) Reminiscences etc. Abel Chapman N° 135

1 The Great Plains Waterfowl Breeding Grounds etc. H.C. Oberholser N° 136

20 Separata por H.C. Oberholser (16) e T.S. Palmer (4) ex: “The Auk” de 1918 a 1928. N° 137a-t

20 Separata ex: American Museum Novitates de 22 de Julho 1924-21-Out.29 N°138a-t

1 Separata Further Notes on Ptilosis – W. De W. Miller ex. Bulleton A.M.N.H. N° 139

1 Separata Nesting Habits of Wagler’s Aropendola (Zarhynchus Wagleri) on Barro Colorado Island. [à caneta:] ex Bulletin A.M.N.H. N° 140

PACOTE N° 8 – 10 Extratos Proceedings of the Biological Society of Washington N°141a-m

2 Sep. ex. Annals and Magazine of Natural History – Oldfield Thomas N°142a-b

1 American Ornithologist’s Union Forty sixth Stated Meeting N° 143

1 Whitney South Sea Expedition-Robert Cushman Murphy N° 144

PACOTE N° 9 – 74 Separata Informações sobre a Avifauna Maranhense (Boletim do Museu Nacional) Emilie Snethlage N° 145

PACOTE N° 10 – 33 Separata – Novas espécies de aves do N.E. do Brasil Dra. Emilie Snethlage – Boletim do Museu Nacional N° 6 – 1925. N° 146

PACOTE N° 11 – 1 A ilha dos Alcatrazes H. Luederwaldt e J. Pinto da Fonseca N° 147

1 Notas biológicas sobre aves brasileiras J. Pinto da Fonseca N° 148

1 Ensaios sobre Ornitologia – Antonio Caetano Guimarães Junior N° 149

1 Uma nova espécie de vespa social do genero Mischocyttarus – J. Pinto Fonseca N° 150

2 Listas dos ninhos de vespas sociais do Brasil, representadas nas coleções do Museu Paulista, J.P. Fonseca N°151a-b

2 As espécies brasileiras do gênero “Laternaria”. N° 152a-b

1 De um novo parasita do cafeeiro (Corthylus affinis, n. sp.) [anotações com caneta:] J. Pinto da Fonseca N° 153

1 Um novo gênero de coccídeo Lecaiinae, (Hemip.). [anotações com caneta:] J. Pinto da Fonseca N° 154

11 Separata – Annals and Magazine of Natural History N° 155a-n

1 A nossa dívida para com os aborígenes F.C. Hoehne. N° 156

1 A importância dos Museus de História Natural, especialmente dos de Zoologia – Rudolf Gliesch N° 157

PACOTE N° 12 – 1 The American Museum School Service N° 158

1 Roosevelt Wild Life Bulletin v.3 N° 3. N° 159

1 Roosevelt Wild Life Bulletin v.4 N° 1. N° 160

1 Catalogue of Birds of the Americas and the adjacente Islands – Ch.B. Cory (Field Museum of H.N. Chicago) v.III N° 161

1 Catalogue of Birds of the Americas and the adjacente Islands – Ch.B. Cory (Field Museum of H.N. Chicago) v.IV N° 162

PACOTE N° 13 – 1 Der Konig Reis – Ernest Roselius N° 163

1 O Torrão Maranhense Raymundo Lopes N° 164

1 Estudo Botânico do Noroeste Philipp von Luetzelburg N° 165

1 Glória sem rumor – Roquette Pinto N° 166

1 Resumo da Geologia do Brasil para acompanhar o mapa Geológico do Brasil. Por John Branner N° 167

1 Arquivo do Instituto Biológico de Defesa Agrícola e Animal N° 1-1928 N° 168

PACOTE N° 14 – 1 O Museu Nacional durante 1921 – Bruno Lobo N° 169

1 O Museu Nacional durante 1922 – Bruno Lobo N° 170

1 Breve notícia sobre A Ornis do Caparaó Pedro Pinto Peixoto Velho N° 171

4 Situação histórico-cultural dos Karayás J.A. Padberg – Drenkpol N°172a-d

PACOTE N° 14 – 1 Resumo de trabalhos executados na Europa em 1924 e 1925. E. Snethlage N° 173

1 Novas espécies de aves do N.E. do Brasil N° 174

1 Morpho Absoloni sp. – Eduardo May N° 175

1 Observação sobre Batrachios brasileiros Dr. Adolpho Lutz N° 176

3 Notas Ornitológicas – Alipio de Miranda Ribeiro N° 177a-c

PACOTE N° 15 – 1 Boletim do Museu Nacional v.III – N°3 N° 178

2 Boletim do Museu Nacional v.IV – N°1 N°179a-b

2 Boletim do Museu Nacional v.IV – N°2 N° 180a-b

1 Boletim do Museu Nacional v.IV – N°3 N° 181

1 Boletim do Museu Nacional v.IV – N°4 N° 182

1 Boletim do Museu Nacional v.V – N°1 N° 183

1 Boletim do Museu Nacional v.V – N°2 N° 184

1 Sep. Nota crítica sobre a Ornis do Itatiaya – Alipio Miranda Ribeiro N° 185

1 Arquivo do Museu Nacional – v.XXIX N° 186

PACOTE N° 16 – 1 Corte de jornal N° 187

1 Jornal “Cinema” N° 188

1 Deutsches Bolksblatt N° 189

1 Boletim Agrícola – 26 de Março 1929 N° 190

1 “The Times” Weekly edition 9 agosto 29. N° 191

2 Tabelle zur Aufnahme südamerikanischer Sprachen. N° 192

1 Envelope sem subscrito N° 193

1 Prospectus. Notes on the Game Birds of Kenya Uganda N° 194

1 Prospectus. Birds of Denmark N° 195

3 Folhas de Illustrated London News, 31 de out. 1925. N° 196

1 Bolsinha de couro com cartões, bússola etc. N° 197

Museu Nacional do Rio de Janeiro, 29 de novembro de 1929.

(a) Eduardo May [com assinatura]

Naturalista contratado.

## Destino dos pertences encontrados em Porto Velho e no Museu Nacional

O material trazido de Porto Velho, somado àquele encontrado na seção de Zoologia, teve diferentes destinos. Parte ficou no Museu Nacional e parte foi encaminhado para a família na Alemanha, por intermédio do secretário consular Hans Spengler e do secretário de legação Friedrich Ried, ambos da “Deutsche Gesandtschaft” [Legação Alemã], no Rio de Janeiro, conforme comprovado em documentos e cartões de visitas:

DEUTSCHE GESANDTSCHAFT

LEGAÇÃO DA ALLEMANHA

Seção Consular

Rio de Janeiro, 3 de fevereiro de 1930

Ilmo. Senhor Professor Dr. Roquette Pinto,

Com referência a nossa conversação a respeito dos bens deixados pela falecida Sra. Dra. Snethlage e arrecadados por esse Museu, tenho a honra de comunicar-lhe que o portador desta, Sr. Hans Spengler, Secretário Consular desta Legação, está autorizado a receber os citados bens e o dinheiro eventualmente deixado pela falecida, bem como a certidão de óbito, pelo que passará o competente recibo.

Quanto aos livros de que me falou, soube da Irmã Anna do Deutsches Frauenheim^10^ que lá foram recebidas somente brochuras procedentes dos Estados Unidos e endereçados à Dra. Snethlage, as quais foram enviadas ao Sr. Professor Snethlage^11^ em Unna, em Westfalen. Talvez haja ainda livros, pertencentes ao Museu, entre os bens arrecadados em Porto Velho, o que o mui ilustre amigo queira mandar verificar.

Continuando sempre a seu inteiro dispor, me subscrevo com elevada consideração.

Friedrich Ried [assinatura]

Secretário de Legação.

Ilmo. Senhor

Professor Dr. Roquette Pinto

D.D. Diretor do Museu Nacional

Rio de Janeiro

O documento seguinte, da mesma data, indica que os bens do espólio foram divididos e entregues aos responsáveis no dia 3 de fevereiro de 1930, no próprio edifício do Museu Nacional:

Rio de Janeiro, [em branco] de [em branco] de 19[em branco]

2ª Via [escrito à tinta]

Aos três dias do mês de fevereiro de 1930, no edifício do Museu Nacional, às duas horas da tarde, na presença do Diretor do Museu Nacional, Prof. Dr. Edgard Roquette Pinto e do Sr. Hans Spengler, Secretário Consular da legação da Alemanha, nesta cidade, foram abertos os volumes que constituíam o espólio da Doutora Emilia Snethlage Naturalista do Museu, falecida em Santo Antonio do Madeira [*sic*], Amazonas, em 25 de novembro de 1929. Separado convenientemente o material pertencente ao Museu Nacional constante da lista abaixo mencionada, foram todos os demais objetos entregues ao Sr. Hans Spengler, que os encerrou em cinco volumes: uma mala de madeira, uma valise de mão, um saco de pano, um caixote de livros e papéis e um caixote com duas espingardas de caça e um guarda-chuva. Os objetos entregues ao Museu Nacional foram: produtos químicos e vidros para coleções, animais conservados, munição de caça, tripé e máquina fotográfica com chassis, um caderno com anotações zoológicas, um maço de folhas manuscritas com descrições de aves, um volume do Boletim do Museu Goeldi (v.VIII), uma espingarda Flaubert, material de dissecção, uma coleção de peles de aves conservadas.

Além dos volumes acima referidos foi entregue ao Sr. Spengler o passaporte da Dra. Emilia Snethlage e uma carta a ela dirigida pelo Sr. Curt Nimnendajú.^12^

Rio de Janeiro 3 de Fevereiro 1930 [à tinta]

Hans Spengler [assinatura]

Roquette Pinto [assinatura]

Dos materiais que pertenciam a Emília Snethlage e que permaneceram no Museu Nacional, embora seja necessário fazer uma análise mais cuidadosa, foram até o momento localizados: a coleção de peles de aves, o caderno com anotações (cadernos de coleta) e algumas folhas manuscritas. As peles de aves devem ter sido imediatamente incorporadas à coleção do Setor de Ornitologia do Museu Nacional, e os documentos aqui analisados encontram-se ali preservados, ao menos, desde 1989 (ver Gonzaga, 1989, p.12), embora não seja possível rastrear quando deram entrada no setor. Possivelmente se perderam no incêndio do Museu Nacional, ocorrido em 2 de setembro de 2018: sua máquina fotográfica, seu tripé, sua espingarda Flaubert e suas fotografias, bem como todos os documentos relativos às suas atividades como funcionária do museu que estavam guardados no Arquivo Histórico do Museu Nacional e que, portanto, ainda eram inéditos.

Após a divisão do espólio entre os membros da Legação Alemã e o Museu Nacional, Friedrich Ried escreveu novamente a Roquette-Pinto para saber se ela teria ainda algo a mais para receber monetariamente:

Rio de Janeiro, 2 de abril de 1930.

Exmo. Amigo e Senhor Dr. Roquette Pinto,

Com referência a nossa última palestra relativo à Senhorita Dra. Snethlage, tomo a liberdade de pedir ao mui ilustre amigo a fineza de comunicar-me se a falecida ainda tem a receber qualquer quantia desse Museu.

Antecipando os melhores agradecimentos, queira aceitar saudações as mais cordiais de quem se subscreve com distinta consideração

amigo admirador

Friedrich Ried [assinatura]

Embora não tenha sido possível encontrar a carta com a resposta para Ried, há um papel, escrito à tinta na parte superior “Diárias da Dra. Snethlage”, que indica os valores que ela deveria receber. Datilografado:

DRA. EMILIE SNETHLAGE

Em excursão de 25^13^ de setembro a 27^14^ de novembro de 1929.

Setembro – 6 dias a 25$000.......150$000

Outubro – 31 dias a 25$000.......775$000

Novembro – 27 dias a 25$000...675$000

Rs. 1:600$000

Com base nesse documento, podemos verificar que a diária de trabalho de Emília Snethlage paga pelo Museu Nacional era de 25 mil réis, e não resta dúvida que esse foi o valor repassado à Legação Alemã, conforme uma carta de resposta de 28 de agosto de 1930:

Rio de Janeiro, 28 de Agosto de 1930

Exmo. senhor,

Tenho a honra de acusar o recebimento da sua carta n.205 de 10 de abril último, na qual informa ter à disposição a quantia de 1:600$000 pertencentes à falecida dra. Snethlage. Peço a especial fineza de mandar depositar essa quantia no Banco Alemão Transatlântico na conta corrente da Legação Alemã.

Com os protestos de minha distinta consideração

[assinatura não identificada]

Secretario de Legação

Exmo. senhor

Dr. prof. Roquette Pinto

M.D. diretor do Museu Nacional

Nesta

## Considerações finais

Por algum motivo, alguns autores divulgaram datas errôneas quanto ao falecimento de Snethlage, como agosto de 1929 (Pinheiro, 2000) e, em especial, 27 de novembro de 1929 (por exemplo Gonzaga, 1989; Pacheco, 2004; Nomura, 2006). Segundo Carvalho (1958, p.39-43), Bertha Lutz, em seu discurso no 139° aniversário do Museu Nacional, indica 29 de novembro de 1929. Não foi possível descobrir onde esses erros iniciaram, porém, em alguns documentos oficiais do próprio Museu Nacional a data de 27 de novembro é por vezes, também, erroneamente informada. Até mesmo em seu último caderno de coleta foi anotada a lápis essa data (Figura 1C). Com os documentos aqui apresentados é, entretanto, inegável que Snethlage faleceu na madrugada do dia 25 de novembro de 1929, no Hotel Brasil, Porto Velho, Rondônia, após ter um infarto do miocárdio.

Os restos mortais de Emília Snethlage continuam desaparecidos, provavelmente por ter ela sido sepultada em túmulo provisório, que não foi perpetuado. As informações aqui apresentadas, porém, poderão auxiliar em sua localização.

A julgar pela sua contribuição às ciências naturais do Brasil, sugere-se que no cemitério dos Inocentes, em Porto Velho, seja erigida uma cruz, réplica da conhecida em fotos, como forma de respeito e homenagem a essa mulher pioneira. Em Porto Velho há um busto, no Memorial Rondon, em homenagem ao seu falecimento, naquela cidade. O Museu Nacional também poderia homenageá-la, por exemplo, com o nome de algum prédio, como ocorre no Museu Paraense Emílio Goeldi (Sanjad, 2019, p.1056).

## Data Availability

Não estão em repositório.

## NOTAS

1 O Setor de Ornitologia do Museu Nacional tem três cadernos de coleta de Emília Snethlage. Esses cadernos são referentes às expedições de: julho de 1925 até janeiro de 1927; março a dezembro de 1927; e abril de 1928 a novembro de 1929. Neles constam as localidades onde coletou e a lista de espécimes coletados por dia, seguindo uma numeração de campo.

2 Essas 120 aves estão tombadas no Setor de Ornitologia do Museu Nacional e em estágio final de análise. As informações relevantes dessa expedição para a ornitologia, como a lista de espécimes e as localidades de coleta, serão publicadas em artigo separado.

3 Antônio Magalhães foi o primeiro diretor brasileiro do Hospital Candelária, empossado em 19 de janeiro de 1928 (Hugo, 1959). O referido hospital foi inaugurado em 1908 para atender os trabalhadores que adoeciam às dezenas durante a construção da Estrada de Ferro Madeira-Mamoré. Ali se hospedaram e trabalharam por um tempo Belisário Penna e Oswaldo Cruz (Cruz, 1910), durante as vistorias sanitárias dos canteiros de obras pelo país (Borzacov, 2007, p.38).

4 Segundo noticiado pelo jornal *Alto Madeira*, de 1 de dezembro de 1929, n.1290, “o seu enterro efectuou-se às 17 horas do mesmo dia, saindo o féretro da igreja do Sagrado Coração de Jesus; sendo bastante concorrido”. Atualmente essa igreja se chama Catedral Sagrado Coração de Jesus e fica na praça Padre João Nicoletti, delimitada pela avenida Carlos Gomes e as ruas José Bonifácio, Dom Pedro II e Gonçalves Dias.

5 Em uma solicitação realizada em fevereiro de 2019, para averiguação do Assento de Óbito de Emília Snethlage nos livros do atual Cartório Godoy, Primeiro Ofício de Notas e Registro Civil, em Porto Velho, o escrevente Ranyer Lopes de Paiva indica, após analisar as entradas de 1º de janeiro de 1929 até 16 de setembro de 1931, “não existir o Assento de Óbito de Emilie Snethlage” naquele cartório. Não foi possível, assim, encontrar o paradeiro do livro que o registra.

6 Embora em estado ruim, esse recorte de jornal ainda permanece entre os documentos encontrados no Setor de Ornitologia do Museu Nacional. Ver nota 4.

7 Embora na nota esteja indicado 25$, em sua última carta Snethlage disse à família que “o hotel com quartos espaçosos e ventilados, rodeado com varandas amplas e protegidas de mosquitos, banheiros, água encanada, luz elétrica, gelo, café da manhã e jantar com várias opções, tudo no maior bom gosto e não tão caro (15 mil réis por dia)” (Sanjad et al., 2013, p.217), assim como no original escrito em alemão pelo sobrinho Emil “(15 Milreis pro Tag)” (Snethlage, 1930, p.131).

8 O Cemitério dos Inocentes foi inaugurado em 28 de julho de 1915. Embora periférico na época, atualmente encontra-se no centro de Porto Velho, tendo sofrido fortemente com o interesse imobiliário. Sua entrada fica na rua Almirante Barroso, cercado pelas ruas Prudente de Morais e Treze de Maio. Foi tombado pelo município, como monumento histórico, pela lei 265 de 26 de dezembro de 1983 (Borzacov, 2007, p.101-102).

9 O mesmo que cabaça, nome do fruto de *Crescentia cujete*, espécie de planta da família *Bignoniaceae*. Muitas vezes cortado ao meio para ser usado como cuia.

10 Deutsches Frauenheim, ou Casa das Mulheres Alemãs. Atualmente Hospital Alemão do Amparo, também conhecido como Hospital do Amparo Feminino, rua Estrela, n.27, bairro do Rio Comprido, Rio de Janeiro. Snethlage passou a residir neste local entre 1925 e 1926, onde permaneceu até seu falecimento.

11 Victor Snethlage (1866-1943), professor em Unna, pai do importante etnólogo Emil-Heinrich Snethlage (1897-1939) (Mere, 2013), era o irmão mais velho, também o mais próximo e querido de Emília (Sanjad et al., 2013).

12 Curt Nimuendajú, nascido Curt Unckel (1883-1945), era de origem alemã e dedicou sua vida estudando os indígenas do Brasil. Trabalhou no Museu Goeldi quando por lá era diretora Emília Snethlage, que foi quem, comprovadamente, o convidou para assumir e organizar a coleção etnográfica do referido museu (Baruja, 2014; Sanjad, 2019). Um trabalho recente indica que Snethlage foi a responsável direta pela inserção de Nimuendajú no meio científico antropológico e etnológico (Sanjad, 2019). A carta aqui encontrada, embora não mencione a data, pode indicar que sua relação com Nimuendajú durou por toda a vida.

13 Data do início da expedição para o rio Madeira.

14
*Vide* discussão sobre essa data.
